# Effects of food-simulating solutions on the surface properties of two CAD/CAM resin composites

**DOI:** 10.4317/jced.59822

**Published:** 2022-10-01

**Authors:** Dina S. Farahat, Noha A. El-Wassefy

**Affiliations:** 1 Assistant Professor, Department of Dental Biomaterials, Faculty of Dentistry, Mansoura University, Mansoura, Egypt; 2 Associate Professor, Department of Dental Biomaterials, Faculty of Dentistry, Mansoura University, Mansoura, Egypt

## Abstract

**Background:**

During clinical service, dental materials could experience chemical degradation due to exposure to different diet components which could affect their functions and longevity. So, the objective of this study was to investigate the effect of food simulating solutions on surface properties of two CAD/CAM dental resin composites.

**Material and Methods:**

Two CAD/CAM composites; a nano-hybrid and a resin nano-ceramic were machined into 2 mm plates then assessed at baseline for their surface roughness and microhardness. Each group was immersed into distilled water, ethanol and methyl ethyl ketone (MEK) for 15 days at 37oC. The surface properties were evaluated after one day, 10 and 15 days of immersion by a surface profilometer and Vickers microhardness tester, and finally the surface morphology was studied using scanning electron microscopy.

**Results:**

At baseline, there was no significant difference in roughness between Teric CAD and Lava Ultimate, however, Lava Ultimate was significantly harder than Tetric CAD. Aging in ethanol had no significant effect on roughness and hardness of both the materials. Yet, Lava Ultimate showed significantly higher roughness and hardness compared to Tetric CAD. Immersion in MEK resulted in a significant increase in roughness of Lava Ultimate at 10 and 15 days. Neverthless, it caused a significant decrease in hardness of Tetric CAD at 10 and 15 days and Lava Ultimate at 10 days. Finally, water immersion caused a significant increase of roughness Tetric CAD.

**Conclusions:**

Exposure to different storage media variably affected the surface properties of CAD/CAM machinable composites. Both materials showed greater stability in surface properties when being immersed in ethanol than MEK. Hence, the surface deterioration suggests the advisability of more research involving increased immersion periods and involvement of thermocycling changes.

** Key words:**Food simulating solutions, chemical degradation, nano-hybrid CAD/CAM composite, resin nano-ceramic CAD/CAM material, surface roughness, micro-hardness, surface morphology.

## Introduction

The development of computer-aided design/computer-assisted manufacturing (CAD/CAM) digital technologies has offered clinicians a variety of treatment options in different fields of dentistry. The use of CAD/CAM allows the fabrication of esthetic restorations with improved mechanical properties in a single visit. Two classes of dental materials are commonly used with CAD/CAM technology: dental ceramics and resin composites. Various materials such as zirconia, feldspathic porcelain, glass ceramics and resin composite blocks are available for use with such technologies for the production of indirect restorations (1,2). Recently, the popularity of resin composite CAD/CAM blocks has increased due to their numerous benefits such as an elastic modulus that is close to that of natural teeth, their ease of milling, the lower abrasiveness to opposing natural teeth, the decreased incidence of chipping and fracture during machining, their lower brittleness compared to glass ceramics and the ease of finishing, polishing and intraoral repair (3).

Composite resin CAD/CAM blocks are manufactured under standardized industrial conditions where polymerization is carried out at high temperatures for longer durations resulting in higher degrees of polymerization compared to direct resin composites. Also, the high pressure applied during manufacturing allows for the use of higher filler fractions and helps produce blocks with homogenous structures and less imperfections (4,5). Consequently, composite resin CAD/CAM blocks exhibit improved biological, physical and mechanical properties with reduced biofilm formation when compared to direct composite resin materials (6). Furthermore, the digital workflow offers a fast manufacturing process that avoids the inherent drawbacks associated with incremental building techniques and polymerization shrinkage in direct restorations and which also allows for the reproduction of the restorations in cases of their failure or loss (7).

A lot of effort has been directed towards the improvement of indirect resin composites including modifications in composition of resin monomers, filler particles and initiation systems, increasing the filler fraction and using different curing methods. Resin composite CAD/CAM blocks may have different microstructures. Polymer infiltrated ceramic networks (PICN) are materials that comprise a porous network of sintered ceramics infiltrated by resin resulting in a hybrid material formed of two interpenetrating networks (8). On the other hand, several resin composite CAD/CAM blocks are based on the conventional filler-resin mixing composite technology. They are composed of resin matrices with dispersed fillers that make use of innovations in resin and filler chemistry in addition to developments in filler loading and polymerization technologies (9).

However, with all the significant advancements in properties of restorative materials, their integrity and durability could still be compromised on exposure to the challenging conditions associated with the oral environment such as chemical, mechanical, biological and physical fluctuations (10). During clinical service, dental materials are exposed to different chemical constituents like salts, acids, alkalis and alcohols that are derived from saliva, dietary or hygienic products or naturally produced by oral microorganisms. Some of these components could have detrimental effects of intra-oral materials including resin composite based materials. Ethanol is an organic solvent that is present in different beverages and has been considered a food simulant by the Food and Drug Administration (FDA). Methyl ethyl ketone (MEK) is also an organic solvent that is naturally found in some fruits and vegetables, dairy products and honey and is also considered a food simulating fluid by the FDA. Water is an important component in human diet that could adversely affect composite resin based restorations. Food simulating fluids have been used to test the durability of different restorative materials by simulating the chemical oral environment. In the artificial aging process, food simulating fluids are used as storage media for the materials under investigation that are simultaneously inspected for signs of degradation (11,12).

The aim of this study was to assess the effect of ethanol and methyl ethyl ketone as food simulating fluids on the surface properties of two composite resin CAD/CAM blocks. The null hypotheses were:

1. There will be no difference in surface roughness and hardness between the two materials at baseline.

2. Food simulating fluids will have no effect on the surface roughness and hardness of the two materials.

3. The time of storage in the food simulating fluids will have no effect on the surface roughness and hardness of the two materials.

## Material and Methods

The composition, manufacturer, batch no and shade of materials used in this study are shown in Table 1.

**Table 1:**
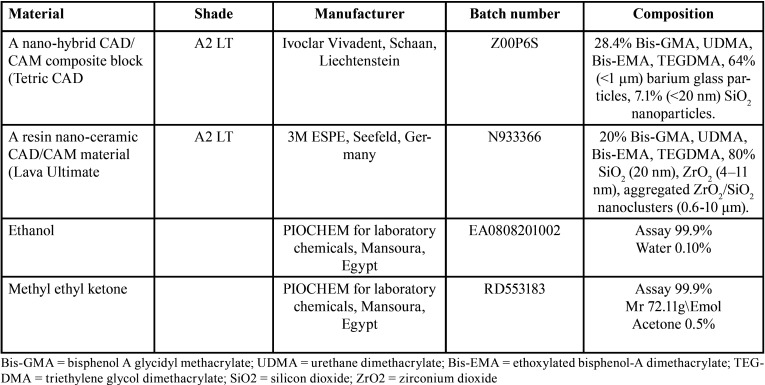
Composition, manufacturer, batch no and shade of materials used in this study.

-Methods 

The blocks were sectioned using a water-cooled low speed diamond wafering blade mounted on a precision saw (Pico 155, Pace Technologies, Tucson, AZ, US), No. PI-BI-0217-004). Specimens were sectioned into plates with 2 mm sections, dimensions were verified using a digital caliper (Electronic stainless steel, Louis Ware, 150 mm / 0-6 inch) with an accuracy of 0.02 mm and a resolution of 0.01 mm. Specimens were smoothed and polished according to manufacturers’ instructions. For Nano-ceramic hybrid material, the specimens were first smoothened and pre-polished by mid grained diamond wheels starting with larger size and then fine grained for high gloss polishing. The Resin nano-ceramic material specimens were first grinded by a medium rubber wheel, and then a soft bristle brush with a polishing agent applied slowly to the surface using a low speed handpiece. The baseline measurements of surface roughness and microhardness were recorded.

-Storage agent immersions

60 specimens were divided into 2 groups of 30 specimens according to the type of materials, and then each group was subdivided into three subgroups of 10 specimens according to the type of storage media; distilled water (served as a control), ethanol, methyl ethyl ketone (MEK). The specimens were then randomly immersed in 10 ml of the storage media in individual glass vials in an incubator at a 37±1ºC and kept under the same conditions for 15 days. After the storage period specimens were taken out of the storage media, rinsed with distilled water and blot dried carefully against filter paper. The surface roughness and microhardness measurements were measured on day one, ten and 15 days, finally surface morphology of specimens was examined on day 15.

Surface Roughness testing

Surface roughness was measured by a stylus contact profilometer (Surftest SJ-210, Mitutoyo, Corp, Kawasaki, Japan) in three directions according to ISO 4278-1997. Three measurements were done by placing the probe over the specimen’s surface. The tracing length was 0.8 mm, at a scanning speed of 0.5 mm/s and the resolution of the recorded data was 0.01 μm. The values of the three readings of each specimen were recorded and the average roughness of each material (Ra) was calculated.

Surface microhardness testing

Surface microhardness was determined using a microhardness tester with a Vickers diamond indenter (Jinan Precision Testing Equipment CO, Model HV-1000ltd, China) at room temperature. Three indentations were made, each being 0.5 mm apart. The indenter was applied against specimens with a load of 200 g for 15 s dwell time.

Vickers hardness number (VHN) was calculated using the following equation:

HV = 2F sin (136/2) /d2 

Where F is the applied force, d is the average length of the indentations’ diagonals and 136 is angle between the face of the diagonal.

-Surface morphology evaluation

The surface morphology was studied using a scanning electron microscope (SEM, JSM-6510LV, JEOL, Tokyo, Japan). First, a representative specimen was selected from each group nad cleaned in an ultrasonic bath for 2 min and then air dried. Then, specimens were gold coated (SPI-MODULETM, SPI Supplies, West Chester, PA, USA) and examined by SEM to assess surface topography using different magnifications.

-Statistical analysis:

Each experiment was repeated in triplicates. Data was presented as means ±  standard deviations (SD). Statistical analyses were performed using SPSS software package for Windows (Version 20.0, SPSS Inc., Chicago, IL). Based on data distribution, three-way analysis of variance (ANOVA) and Tukey’s post hoc tests were used for statistical comparisons among groups. Differences were considered significant when *p*< 0.05.

## Results

The Shapiro-Wilk test revealed that all data followed a normal distribution pattern in all research groups. Therefore, a parametric three-way analysis of variance (ANOVA) was conducted first, followed by post-hoc tukey test. All tests were measured at baseline, after one day, 10 days and after 15 days. The outcomes of ANOVA test revealed that “type of materials”, food simulating solutions, “immersion period” significantly affected average roughness and Vickers microhardness results (*p*<0.05).

1. Roughness results:

Surface roughness was significantly affected by material and immersion time (*p*<0.0001) and by food simulating solutions *p*=0.0043. Results of surface roughness testing are shown in Tables 2,3. At baseline, there was no significant difference in surface roughness between Tetric CAD and Lava Ultimate (*p*=0.15). After aging in ethanol, there were no statistically significant differences between surface roughness at baseline and surface roughness after storage for one, 10 and 15 days for both Lava Ultimate and Tetric CAD. However, surface roughness of Lava Ultimate was significantly higher than that of Tetric CAD after 15 days of immersion.

**Table 2:**
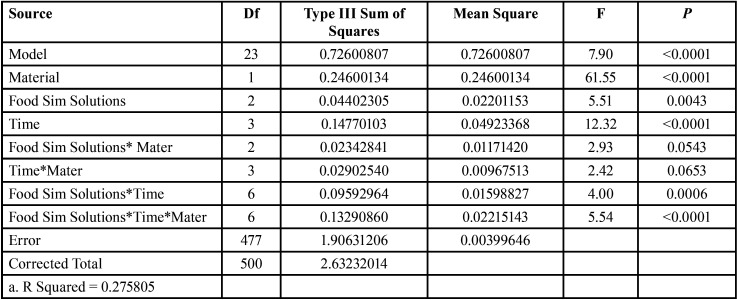
Three way ANOVA showing the effect of Groups and Time on roughness measurements(um).

**Table 3:**
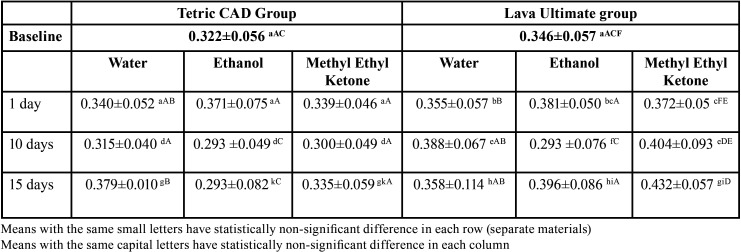
The means and standard deviations of roughness values (um) of Tertric CAD and Lava Ultima materials at baseline, after immersion in water, ethanol, methyl ethyl ketone groups, at one day, 10 days and 15 days of immersion.

After immersion in MEK, surface roughness increased significantly from baseline for Lava Ultimate after 10 and 15 days (*p*=0.0001 and *p*=0.028 respectively), but there were no significant differences in surface roughness for Tetric CAD at all timepoints from baseline. Yet, surface roughness of Lava Ultimate was significantly higher than that of Tetric CAD after 15 days of MEK immersion (*p*<0.0001). Immersion in water significantly increased surface roughness for Tetric CAD after 15 days compared to baseline (*p*=0.0018) . However, no significant effect was found for immersion in water on surface roughness of Lava Ultimate at all aging times.

2. Hardness results:

Surface micro-hardness was significantly affected by material (*p*<0.0001), immersion time( *p*=0.0002) and by food simulating solutions (0.0026) as shown by the results of the Three-way ANOVA test. Results of surface micro-hardness testing are shown in Tables 4,5. At baseline, Tetric CAD had significantly lower hardness than Lava Ultimate (*p*<0.0001). After immersion in ethanol, hardness values recorded at all timepoints were not significantly different from baseline for both the materials. Following aging in MEK, micro-hardness significantly decreased from baseline for Tetric CAD at 10 and 15 days (*p*<0.0001), and for Lava Ultimate at 10 days. Immersion in water significantly increased surface hardness for Lava Ultimate after 10 days compared to baseline (*p*=0.0084). Nevertheless, no significant effect was found for immersion in water on the surface hardness of Tetric CAD at all immersion times (*p*=0.1617).At all periods of immersion in distilled water, ethanol, and methyl ethyl ketone, the Lava group had significantly higher surface hardness values compared to the Tetric group, *p*<0.0001.

**Table 4:**
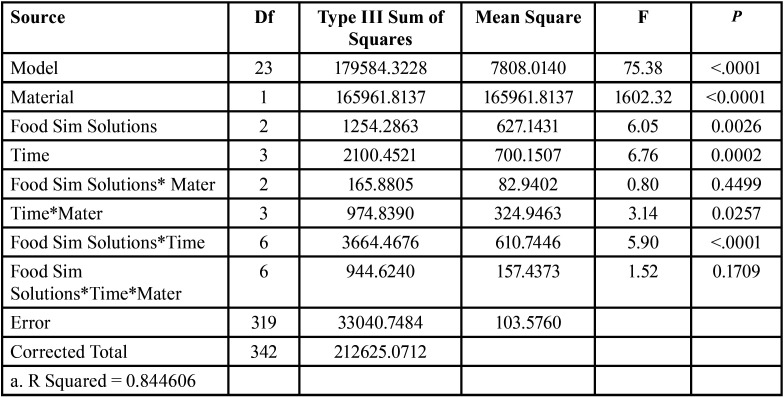
Three way ANOVA showing the Effect of Groups and Time on hardness measurements (kg.mm-2).

**Table 5:**
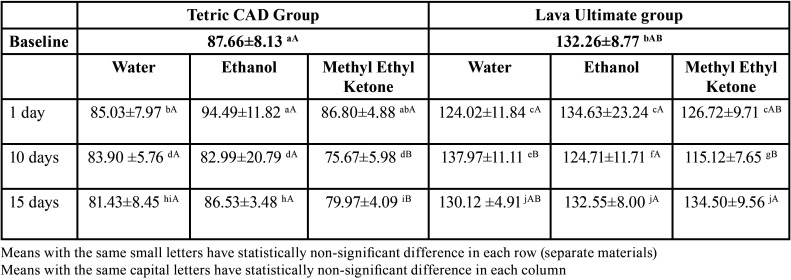
The means and standard deviations of hardness values (kg.mm-2) of Tertric CAD and Lava Ultima materials at baseline, after immersion in water, ethanol , methyl ethyl ketone groups, at one day, 10 days and 15 days of immersion.

-Surface morphology evaluation

**Figure 1:**
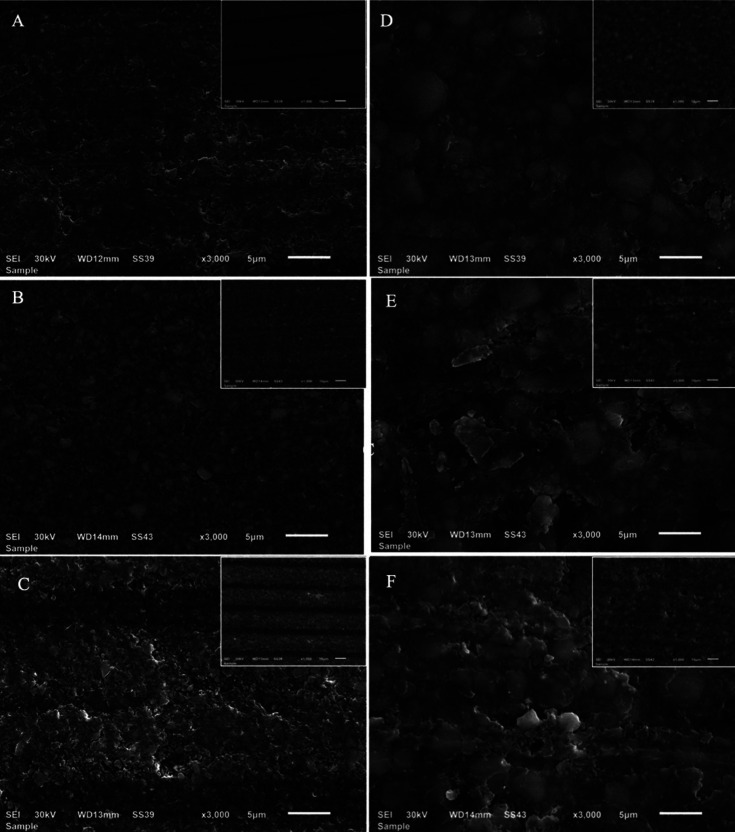
Scanning electron photomicrograph of Tetric CAD (left column) and Lava Ultimate (right column) specimens immersed for 15 days in storage solutions at X 3000 and X 1000 (smaller image). A,B,C photomicrographs of Tetric specimens after immersion for 15 days in distilled water, ethanol, and methyl ethyl ketone respectively. D,E,F photomicrographs of Lava Ultimate specimens after immersion for 15 days in distilled water, ethanol, methyl ethyl ketone respectively.

**Figure 2:**
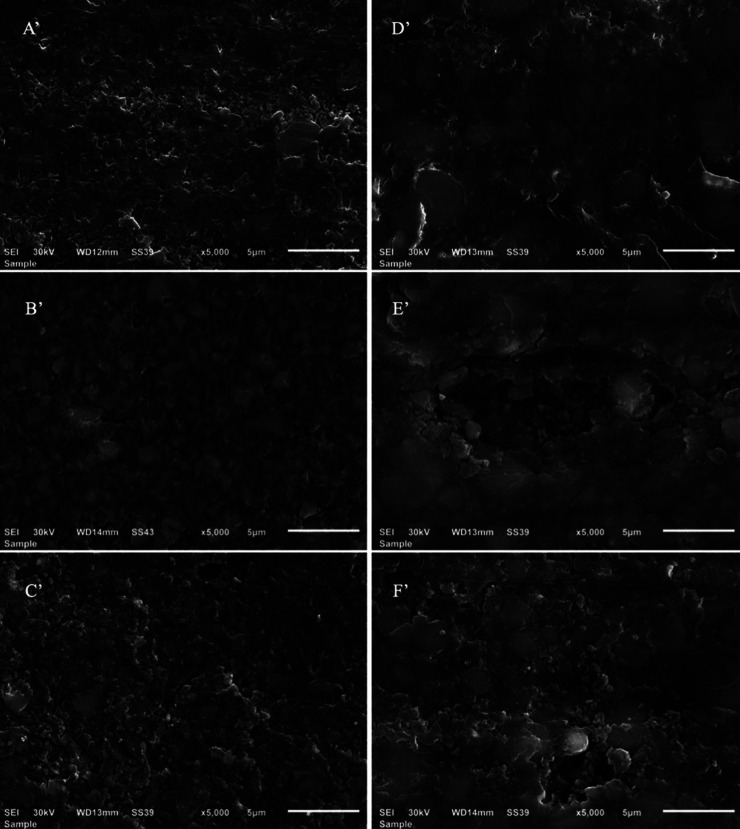
Scanning electron photomicrographs of Tetric CAD (left column) and Lava Ultimate (right column) specimens immersed for 15 days in storage solutions at X 5000. A’,B’,C’ photomicrographs of Tetric specimens after immersion for 15 days in distilled water, ethanol, methyl ethyl ketone respectively. D’,E’,F’ photomicrographs of Lava Ultimate specimens after immersion for 15 days in distilled water, ethanol, methyl ethyl ketone respectively.

Figures 1 and 2 show scanning electron photomicrographs of Tetric and Lava Groups for specimens immersed for 15 days in storage solutions (X 1000, 3000 and 5000). Photomicrograph of Tetric specimens immersed in distilled water show a well condensed polymer matrix with filler particles and negligible porosity (Figs. 1A,2A’); photomicrographs of Tetric specimens immersed in ethanol show fewer polymer matrices with well prominent filler particles and very few narrow porosities (Figs. 1B, 2B’); photomicrographs of Tetric specimens immersed in methyl ethyl ketone show fewer polymer matrices with well prominent filler particles and few smaller sized porosities (Figs. 1C, 2C’).

Photomicrographs of Lava specimens immersed in distilled water show polymer matrices with multimodal filler particle size distribution and fewer pinpoint porosities (Figs. 1D,2D’); photomicrographs of Lava specimens immersed in ethanol show less polymer matrices on the top surface with well prominent filler particles of heterogeneous size distribution and large irregular size deepened porosities (Figs. 1E,2E’); photomicrographs of Lava specimens immersed in methyl ethyl ketone show fewer polymer matrices on the top surface with well prominent filler particles of heterogeneous size distribution and large sized porosity (Figs. 1F,2F’).

## Discussion

Besides distilled water, ethanol and MEK are standard storage media used for aging research. These organic solvents mimic the effect of food and dental cleaning products on the physico-mechanical properties of dental resins (13). In the current study, the effect of food simulating fluids on the surface roughness and hardness of two resin composite CAD/CAM blocks was evaluated. At baseline, the surface roughness of both materials was not significantly different, however, Lava Ultimate showed significantly higher hardness values so the first null hypothesis had to be rejected. Immersion in water, ethanol and methyl ethyl ketone had a significant effect on surface roughness and hardness of the two CAD/CAM materials at some timepoints during the study so the second and third null hypotheses were rejected.

The effect of aging in food simulating fluids on the roughness and hardness of composite and polyacid modified composite restorative materials was studied by Yap *et al*. They found that there was no significant difference in roughness and hardness of most of the materials after immersion in ethanol (14). The effect of water, methyl ethyl ketone (MEK) and ethanol as food simulating liquids on three CAD/CAM polymer composites was also investigated. The polymer composites included two different fiber-reinforced composites and a reinforced PEEK. It was reported that MEK and ethanol caused more evident changes in the surface properties compared to water. Fibre-reinforced composite displayed more pronounced deterioration in MEK whereas PEEK showed slight changes in ethanol (15). Badra *et al*. investigated the effect of different beverages on the microhardness and surface roughness of resin composites. They stated that after immersion in an ethanol containing beverage, there was no significant difference in microhardness after 7 days, but hardness significantly increased after 30 days then decreased significantly after 60 days. Furthermore, roughness increased after 7 days then significantly decreased after 30 and 60 days (12).

Surface roughness is an important characteristic that could impact the aesthetics and longevity of tooth colored restorations. Chemical degradation in the oral cavity can increase surface roughness thus promoting plaque adhesion with subsequent discoloration, gingival inflammation, and secondary caries (16). In this study, a contact profilometer was used to assess surface roughness of both types of resin composite CAD/CAM blocks after immersion in three different food simulating fluids. There were no significant differences in roughness values for both types of materials from baseline after immersion in ethanol at all timepoints, yet the recorded roughness of LAVA ultimate was significantly higher than that of Tetric CAD. On storage in fluids, resin based matrices tend to absorb the fluids whose molecules percolate the network and diffuse between polymer chains. An increase in distance between chains results in swelling and plasticization of the material. The plasticizing effect of ethanol on dental composites was reported in several investigations (17-20). It was suggested that the increased plasticization could render surface flaws less severe (21,22).

However, the dimensional changes due to swelling of resin based polymers can cause stresses on the bond between the matrix and filler leading to its failure and the formation microcracks within the matrix. The subsequent debonding of the inorganic filler particles will result in porosities that would be expected to increase surface roughness (23). Ferracane and Marker observed cracking in resin composite matrix and resin filler interface after immersion in ethanol using SEM (24). This could explain the significantly higher roughness of Lava Ultimate compared to Tetric CAD blocks after immersion in ethanol. Both the materials utilize nanohybrid filler technology. Tetric CAD contains silica nanoparticles (<20 nm) and barium glass particles with a size < 1 μm, whereas Lava Ultimate comprises silica and zirconia nanomers (6-20 nm) and silica and zirconia aggregated nanoclusters (6-10 μm). It could be postulated that the loss of larger sized nanoclusters in Lava Ultimate (Figs. 1E,2E’), due to filler debonding, would lead to an increase in surface roughness compared to the loss of smaller barium and silica that might occur in Tetric CAD as observed in Figs. 1B,2B’. Furthermore, Tetric CAD comprises a higher percentage of resin matrix (28.4%) than Lava Ultimate (20%), so it would be expected to be more prone to plasticization caused by ethanol (25).

The sorption and solubility properties of polydimethacrylate based polymers is affected by several factors such as the presence of reinforcing fillers, the resin fraction, the hydrophilicity of the polymer, the cross-linking of the polymer network and most importantly the difference between the solubility parameters of the polymer and that of the solvent (26). The square root of the cohesive energy density of a solvent is used to calculate its solubility parameter (25). The smaller the difference in solubility parameters of the polymer and the solvent, the greater the solvent uptake and polymer solubility. The solubility parameters of ethanol and MEK are close to that of the dimethacrylate monomers commonly used in dental resin composites. After immersion in MEK, both the materials in this study showed an increase in surface roughness compared to the baseline being significant only for Lava Ultimate. The solubility parameter of dimethacrylate based resins (18.6 δ/MPa1/2) is closer to that of MEK (19.3 δ/MPa1/2) than ethanol (26.2 δ/MPa1/2) and more different than water (48 δ/MPa1/2). Accordingly, the resin degradation would be expected to be more evident with MEK than with ethanol (27,28). The scanning electron micrographs of Tetric CAD specimens (Figs. 1C,2C’) showed a surface with exposed inorganic fillers with increased resin degradation which could be due to the increased solubility of resin in MEK. On the other hand, Lava Ultimate showed an increase in roughness at all recorded timepoints after MEK immersion and the highest roughness value among the specimens after immersion in the three liquids for 15 days.

Immersion in water affected the surface roughness of Tetric CAD in which roughness values increased significantly from the baseline. Sideridou *et al*. described the degradation of direct composite surfaces due to water absorption. Water uptake can lead to softening of the resin matrix, hydrolysis of the silane coupling agent, microcrack formation, resin degradation and filler debonding (21). Hence, aging of resin composites in different food simulating fluids can yield materials that are soft and flexible due to fluid absorption at first. Yet, over time, material property changes occur due to chemical degradation caused by hydrolysis, stresses associated with swelling, and leaching (23,29).

The surface microhardness of a material indicates how it resists surface penetration and indentations, increasing the surface hardness, indicates the higher the mechanical properties. Chemical degradation of these CAD/CAM composites after immersion in food simulating fluids, as evidenced by a decrease in hardness, is due to diffusion of solvent molecules between filler particles and matrix. This resulted in fillers dislodgement from the polymer matrix. Diffusion of solvent molecules within the matrix structure also made it softer and less wear-resistant (30). The average surface hardness of Lava Ultimate was significantly higher than Tetric CAD at baseline level; this might be attributed to its higher percentage of filler loading, type of filler (zirconia) and their intimate compaction due to their nanocluster composition aggregation (31). The increase in filler content was shown to result in lower water absorption, and consequently reduced surface degradation (31). Water absorption and hydrolytic degradation of the filler surface can predispose to filler/matrix cracking which has been shown to adversely affect the mechanical properties of composites (32). In this study, the surface microhardness of Tetric CAD decreased in-significantly than the baseline when being immersed in distilled water and ethanol solutions for 15 days, while significantly decreased in MEK solution. The decrease in hardness in MEK solution can be attributed to its solubility parameter that is closer to the resin and thus more resin degradation occurred, as noted in Figs. 1C,2C’).

On the other hand, the surface microhardness of Lava Ultimate group decreased in-significantly than the baseline when being immersed in distilled water and ethanol for 15 days. However, it increased in-significantly after being immersed in MEK, this might be due to the higher fillers loading and their nanoclusters distributions, that was maintained and highly compacted as seen in Figs. 1F,2F’), the little effect of resin matrix dissolution as the Lava ultimate has 20% resin content.

## Conclusions

The surface properties of CAD/CAM machinable composites were variably impacted by the exposure to different aging media. Both materials showed greater stability in surface properties when being immersed in ethanol than MEK. Hence, the surface degradation implies the advisability of more research involving increased immersion periods and involvement of thermocycling changes.
